# Probiotic *Escherichia coli* Nissle 1917 Expressing Elafin Protects Against Inflammation and Restores the Gut Microbiota

**DOI:** 10.3389/fmicb.2022.819336

**Published:** 2022-05-06

**Authors:** Guigen Teng, Zilin Liu, Yun Liu, Ting Wu, Yun Dai, Huahong Wang, Weihong Wang

**Affiliations:** Departments of Gastroenterology, Peking University First Hospital, Beijing, China

**Keywords:** Elafin, EcN, gut microbiota, inflammatory bowel disease, intestinal epithelial barrier

## Abstract

Intestinal mucosal inflammation and epithelial barrier dysfunction have been implicated as pathological factors in inflammatory bowel disease (IBD). An emerging area of IBD research focuses on probiotics. The probiotic *Escherichia coli* Nissle 1917 (EcN) is an excellent choice for engineering therapeutic microbes. Elafin is an endogenous specific inhibitor of neutrophil elastase (NE) and proteinase 3, and we previously found Elafin can effectively suppress the development of colitis. Here, we genetically engineered EcN to deliver Elafin (EcN-Elafin) directly to the colonic mucosa and explored the protective effects of EcN-Elafin against colitis in mice. EcN-Elafin significantly alleviated dextran sodium sulfate (DSS) induced colitis. Compared with wild-type EcN, oral administration of EcN-Elafin displayed better effects on loss of weight, colon length shortening, elevated expression of myeloperoxidase (MPO), and proinflammatory cytokines and chemokine in colonic tissues. In addition, EcN-Elafin restored the expression and distribution of tight junction protein ZO-1 in colonic tissues back to normal. In a damaged colonic epithelial model utilizing Caco-2 cells stimulated with TNF-α, EcN-Elafin efficiently downregulated the activation level of NF-κB signaling. EcN-Elafin was also found to have restored the dysbiosis in gut caused by DSS administration. Moreover, EcN-Elafin significantly enhanced the concentrations of butyrate and valerate in the gut lumen. Thus, our findings demonstrated that EcN*-*Elafin enhanced the colonic epithelial barrier, promoted the resolution of inflammation, modulated the gut microbiota, and elevated concentrations of short-chain fatty acids (SCFAs) in the gut. EcN-Elafin may be a potential therapeutic method for IBD.

## Introduction

Inflammatory bowel disease (IBD), which includes ulcerative colitis (UC) and Crohn’s disease (CD), is a chronic relapsing disorder that affects the alimentary tract (Ng et al., 2017; Ma et al., 2019). The incidence of IBD is high in developed countries and has been increasing over the past decades in newly industrialized countries (Neurath, 2019). IBD is characterized by its progressive and destructive nature and may cause various complications, such as stenosis, fistulas, and colitis-associated cancer. Thus, effective therapeutic approaches are of high clinical relevance in IBD patients.

The pathogenesis of IBD is multifactorial, including genetic predisposition, epithelial barrier defects, dysregulated immune responses, and microbial dysbiosis (Neurath, 2019). An impaired intestinal barrier facilitates the diffusion of pathogens and noxious antigens from the lumen into the internal milieu, therefore initiating inflammatory responses. In IBD, inflammation might arise from abnormal host-microbial interactions that lead to the disruption of intestinal homeostasis. Restoring the intestinal barrier and microflora may improve the efficacy of IBD treatment.

Elafin is a low molecular weight peptide originally identified as a protease inhibitor in the lung and skin (Sallenave and Ryle, 1991; Shanahan and Collins, 2010) that specifically inhibits neutrophil elastase (NE) and proteinase 3 (Sallenave, 2010). The anti-inflammatory effect of Elafin in respiratory disorders has also been well established (Verrier et al., 2012). Over the past decade, an important body of studies have highlighted the importance of proteolytic balance disruption in the pathogenesis of IBD (Solà-Tapias et al., 2020). We previously showed that Elafin expression was significantly downregulated in the colonic mucosa of IBD patients, which was correlated with an elevated disease activity index (Zhang et al., 2017). Moreover, overexpressed Elafin in mice is able to enhance the intestinal epithelial barrier and protect against experimental colitis, suggesting that exogenous Elafin could be a potential agent for the treatment of IBD (Motta et al., 2011). However, peptide drugs are unstable in the gastrointestinal tract and are unable to reach the target site through oral administration. Thus, an efficient system for delivering Elafin to the gut epithelial surface is required. As probiotics exert protective effects on intestinal epithelial function (Suez et al., 2020), they might be an appropriate vector for therapeutic peptides to facilitate their anti-inflammatory effect in the gut.

Several nonpathogenic bacteria especially species from *Lactococcus* and *Escherichia* are good carriers for delivering therapeutics to the gut (Praveschotinunt et al., 1917; Steidler et al., 2000; Motta et al., 2012; Hanson et al., 2014). Compared with other probiotics, *Escherichia coli* Nissle 1917 (EcN) is a well-established bacterium for genetic modification (Rembacken et al., 1999; Kruis, 2004; Coqueiro et al., 2019). Thus, EcN meets all requirements for a therapeutic agent and is well recognized as a safe probiotic for human use. As a popular engineering chassis for delivering therapeutic bioagents to the gut, EcN has several superiorities: (i) EcN is safe to be use in humans, (ii) EcN is compatible with common genetic engineering techniques applied in bacteria, and (iii) EcN is a facultative anaerobic bacterium which means it can grow under aerobic, microaerobic, and anaerobic conditions (Coqueiro et al., 2019; Yan et al., 2021). Although EcN has made some progress in treating IBD, the low overall efficacy and high relapse rates have limited its application as first-line treatment for IBD (Scaldaferri et al., 2016). In this study, we aimed to construct Elafin-expressing EcN and determine its therapeutic effects on experimental colitis, with a particular focus on its impact on the intestinal epithelial barrier function and microbiota composition.

## Materials and Methods

### Construction of Recombinant Elafin-Expressing EcN

EcN used in this study was provided by Ardeypharm GmbH (Herdecke, Germany). Plasmid pGEX-4 T-3 (GE Healthcare Life Sciences) was utilized as vector to introduce Elafin DNA into EcN. Gene encoding for Elafin was amplified *via* high-fidelity polymerase chain reaction (PCR) from a human Elafin cDNA plasmid. Primers were designed to eliminate the sequence encoding for signal peptide (1–20 amino acids). The pGEX-4 T-3 needs IPTG or lactose to induce the replication of the plasmid which is not suitable for *in-situ* experiment in a colitis model for the reason that IPTG or lactose must be added to the drinking water to induce the expression of Elafin. In order to construct the recombinant bacteria which can express Elafin without induction, we removed partial sequences of LacI (354 nucleobases encoding for the 153–271 amino acids were removed) in pGEX-4 T-3 and the resulting plasmid fused with Elafin cDNA was then transferred into EcN cell.

### Bacteria Culture and Bacterial Extracts Preparation

EcN-WT and EcN-Elafin bacterial cells were cultured at 37°C in Luria-Bertani broth (LB) medium with intense rotation (210 rpm). Bacterial growth was monitored by measuring the optical density at 600 nm (OD600). After the bacteria had grown to the required concentration, the bacterial suspension was then centrifuged at 5000 rpm for 10 min, washed, and resuspended in the corresponding volume of sterile PBS to obtain 5 × 10^9^ colony-forming units (CFU) for oral gavage in mice. For bacterial extract preparation, EcN-WT and EcN-Elafin bacteria resuspended in 5 ml of cold PBS were subjected to ultrasonic homogenizers, cell debris was removed by centrifugation, and the supernatant was filtered through a 2 μm filter and total protein concentrations were determined by the BCA method.

### Experimental Animal and Induction of Chronic Colitis

**Figure 1:**
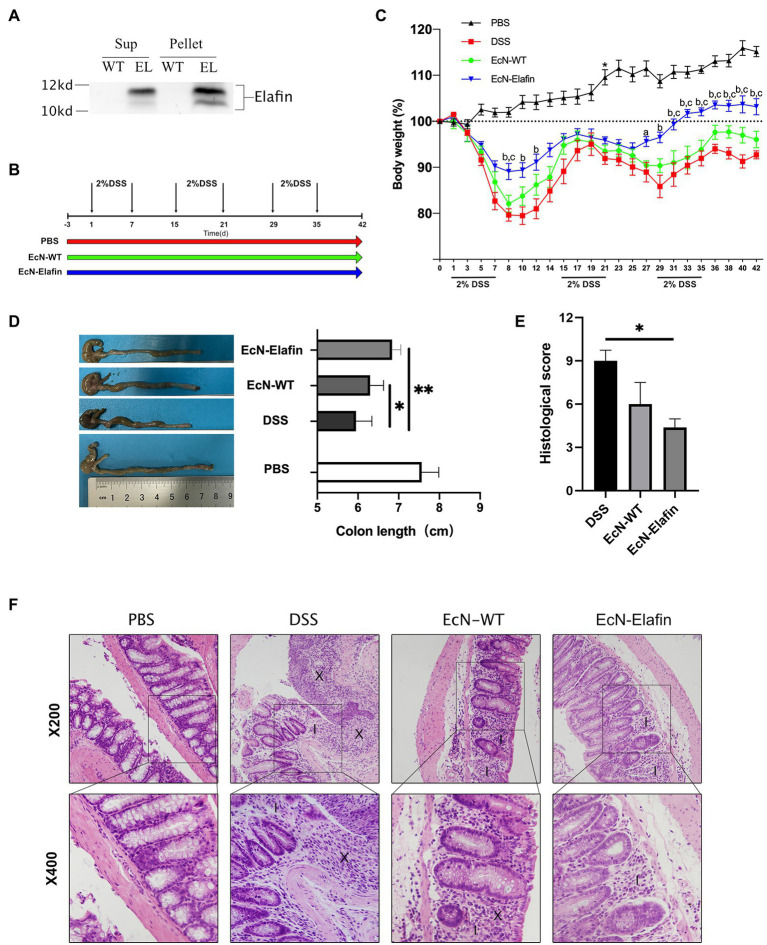
EcN-Elafin ameliorated DSS-induced chronic colitis in mice. **(A)** Detection of Elafin in supernatants and bacterial homogenates of EcN-WT and EcN-Elafin using Western blotting. **(B)** Schematic of administration schedule. EcN-WT (10^9^ CFU) and EcN-Elafin (10^9^ CFU) were administered orally once daily. **(C)** Body weight changes of the mice in each group after DSS administration. ^a^*p <* 0.05 and ^b^*p* < 0.01 vs. DSS group; ^c^*p* < 0.05 vs. EcN-WT group. **(D)** Representative photo of colons and lengths of the colons in each group. **(E)** Histopathological scores of colonic sections in each group. **(F)** Representative photos of H&E-stained sections of colonic tissues from each group. Image markers indicate loss of crypt and goblet cell depletion (X), immune cell infiltration (I). *n* = 6–8 in each group. The data are presented as the mean ± SEM. ^*^p < 0.05; ^**^p < 0.01.

Male C57BL6/J mice (8–9 weeks old) obtained from Vital River Inc. (Beijing, China) were housed under SPF conditions and under a 12 h light/dark cycle at room temperature, and the mice were given free access to sterile food and water. The mice were allowed at least 1 week of acclimatization to the facility before any experiments. All the experimental procedures were approved by the Animal Experimentation Ethics Committee of Peking University First Hospital and were conducted in compliance with guidelines of the China Laboratory Animal Management Committee. The mice were randomly divided into four groups: the control group (PBS group, *n* = 6); colitis group (DSS group, *n* = 8); colitis group with EcN-WT administration (EcN-WT group, *n* = 6); and colitis group with EcN-Elafin administration (EcN-Elafin group, *n* = 6). Colitis in mice was induced *via* treatment with DSS dissolved in drinking water. The mice in all four groups except the PBS group received 2% DSS (w/v) treatment for 7 days followed by regular drinking water for 7 days, and this protocol repeated for another two rounds to generate DSS-induced mice model of chronic colitis (schematic outlined in Figure 1B). The colon of each mouse was removed and its length was measured. Snap froze colonic tissue with a length of 1 cm long starting from the anus in liquid nitrogen and store at −80°C until RNA extraction and cytokine quantification. The rest of the colon was fixed in 4% paraformaldehyde for colitis histological analysis using Swiss-rolling technique as described previously (Bialkowska et al., 2016).

### Histopathological and Immunohistochemical Analyses

For histopathologic analysis, 4% paraformaldehyde fixed colon sample was embedded in paraffin, sectioned (4 μm), and stained with hematoxylin and eosin (H&E). Histological scoring was performed in a blinded fashion by an investigator. Colitis scoring was made as a combined score for severity of inflammation (0 = normal; 1 = slight; 2 = moderate; and 3 = severe), depth of injury (0 = normal; 1 = mucosa; 2 = mucosa and submucosa; and 3 = transmural), crypt (damage: 0 = normal; 1 = basal one-third damaged; 2 = basal two-thirds damaged; and 3 = only surface epithelium intact), and percent of involved area (0 = normal; 1 = 1–25%; 2 = 26–50%; 3 = 51–75%; and 4 = 76–100%). For immunohistochemistry, the expression of myeloperoxidase (MPO) and ZO-1 was detected using rabbit anti-MPO monoclonal antibody (Abcam) and rabbit anti-ZO-1 monoclonal antibody (Invitrogen), respectively. Integrated optical density (IOD) values were measured by Image-Pro Plus 6.0 image processing software (Media Cybernetics). Five fields of vision (at magnification of ×400) per section (two sections per specimen) were used for DSS colitis histological scoring and MPO expression quantification.

### Quantitative Real-Time PCR

For gene expression analysis, colonic tissues were homogenized in Trizol reagent (Invitrogen). Total RNA was extracted and reverse transcribed into cDNA using high capacity cDNA Reverse Transcription Kits (Invitrogen), and qPCR assays were performed with 7,500 Fast Real-Time PCR System (Applied Biosystems) using Power SYBR Green PCR Master Mix (Applied Biosystems). Expression of GAPDH was used as an endogenous control. Primers used for RT-qPCR are listed 5′–3′ in Table 1.

**Table 1:** Mouse primers used for RT-qPCR.

Primer Name	Sequence (5′-3′)
IL-6-F	CTGCAAGAGACTTCCATCCAG
IL-6-R	AGTGGTATAGACAGGTCTGTTGG
TNF-α-F	CAGGCGGTGCCTATGTCTC
TNF-α-R	CGATCACCCCGAAGTTCAGTAG
CXCL-1-F	ACTGCACCCAAACCGAAGTC
CXCL-1-R	TGGGGACACCTTTTAGCATCTT
GAPDH-F	AGGTCGGTGTGAACGGATTTG
GAPDH-R	TGTAGACCATGTAGTTGAGGTCA

### Cytokine and Chemokine Analysis

The levels of IL-6, TNF-α, and CXCL-1 in mice colonic tissues were determined by ELISA using commercial kits from Invitrogen (Catalog No. 88-7, 064-22, 88-7, 324-22, and EMCXCL1) according to the manufacturer’s instructions. The results are presented as picograms per milligram of total protein (pg/mg proteins).

### Caco-2 Cell Culture and Western Blotting Analysis

Caco-2 cells were maintained at 37°C in Dulbecco’s modified Eagle’s medium (DMEM) with high glucose and an extra addition of 50 U/ml penicillin, 50 U/ml streptomycin, and 10% fetal bovine serum. For cell stimulation, 7.5 ng/ml TNF-α (PeproTech) was added to medium with or without 50 ng or 100 ng of either EcN-WT or EcN-Elafin extracts to Caco-2 cells. After 30 min incubation at 37°C, the whole protein of cells was extracted as described previously (Ye et al., 2011). Briefly, Caco-2 monolayers were washed with freezing PBS and then, adequate volume of cold RIPA buffer (Thermo Scientific) containing protease and phosphatase inhibitor (Thermo Scientific) was added. Cells were kept on ice for 5 min with occasionally swirling. Then, cell lysate was collected using a cell scraper and transferred to microcentrifuge tube. The whole protein of cell lysate was obtained by centrifugation at 16000 rpm for 10 min. The protein concentrations were determined using a BCA kit (Thermo Scientific). Caco-2 cell lysates containing equal quantities of whole proteins (40 μg) were then electrophoresed in 10% polyacrylamide gels. Subsequently, the proteins were transferred into a PVDF membrane (Millipore). The membrane was blocked for non-specific binding for 1 h (5% BSA in TBST buffer) at room temperature and then incubated overnight at 4°C with rabbit anti-glyceraldehyde-3-phosphate dehydrogenase monoclonal antibody, rabbit anti-phospho-IκBα (Ser32) monoclonal antibody, mouse anti-IκBα monoclonal antibody, rabbit anti-phospho-IKKβ (Ser181) monoclonal antibody, rabbit anti-IKKβ monoclonal antibody, rabbit anti-phospho-NF-κB P65 (Ser536) monoclonal antibody, and rabbit anti-NF-κB P65 monoclonal antibody (all from Cell Signaling Technology).

### 16S rDNA Sequencing and Short-Chain Fatty Acid Quantification

With the harvest of intestine in each mouse, fecal samples were collected with sterile forceps. Total genomic DNA was extracted from fecal samples using the QIAamp DNA Stool Mini Kit (Qiagen, Germany) according to the manufacturer’s protocol. Bacterial primers 341F (5’-CCTACGGGAGGCAGCAG-3′) and 806R (5’GGACTACHVGGGTWTCTAAT-3′) targeting the V3-V4 hyper-variable region (HVR) of 16S rDNA gene was used for PCR amplification. Then, these amplicons were sequenced on NovaSeq PE250 platform (Illumina) for paired-end reads. The raw sequencing data were assembled using Pandaseq (Masella et al., 2012) and then filtered to get clean reads. Afterward, clean reads were demultiplexed according to the sample-specific barcodes, assigned to operational taxonomic units (OTUs) at a minimum of 97% sequence similarity using USEARCH (Edgar, 2013; V7.0.1090) and then mapped to the 16S rDNA database (RDP)^1^ to get the mapped reads at last. Prior to downstream analysis, we removed the singleton OTUs to reduce potential errors in sequencing. For metabolite extraction, 50 mg of fecal sample was placed into 2 ml EP tubes, add 50 μl of phosphoric acid, 400 μl ethyl ether, and 100 μl 4-methylvaleric acid (125 μg/ml) used as the internal standard. This mixture was vortexed for 1 min, then centrifuged at 12000 rpm for 10 min. SCFAs in the supernatant were sequentially quantified by Thermo TRACE 1310-ISQ LT GC–MS system (Thermo Fisher).

### Statistical Analysis

The data were analyzed using Prism 7 (GraphPad Software), and statistical significance between groups was determined using one-way ANOVA followed by Tukey’s multiple comparisons. *p* < 0.05 were considered statistically significant. For SCFAs data analysis among four group, Kruskal-Wallis test was used.

## Results

### Genetically Engineered EcN Is Capable of Expressing Elafin

In order to deliver Elafin to the surface of colonic surface, we created plasmid-based recombinant EcN encoding for human Elafin protein. As shown in Figure 1A, concentrated bacterial culture supernatants and bacterial pellet homogenates were subjected to Western blotting analysis. Bands at 12 kDa corresponding to the Elafin protein were detected in both the bacterial culture supernatants and pellet lysates of genetically engineered EcN. No signal was detected in the supernatants of the bacterial culture or pellet homogenates of the wild-type EcN strain.

### EcN-Elafin Ameliorates Chronic DSS-Induced Colitis

Based on the ability of the engineered EcN to constitutively express Elafin *in vitro*, we sought to investigate its efficacy in treating DSS-induced chronic colitis in mice. We used a model of chronic DSS-induced mouse colitis that can mimic the relapsing course of disease and chronic forms of intestinal inflammation that resemble some features in UC patients (Figure 1B). The mice received wild-type EcN and EcN-Elafin by oral gavage for three consecutive days before DSS administration and throughout the whole experiment until the day before they were sacrificed. As shown in Figure 1C, challenge with DSS has caused severe intestinal inflammation, as shown by weight loss compared with the PBS group. Mice in EcN-Elafin group showed significantly improved weight loss over the whole three rounds of DSS exposure and almost returned to the same level as the PBS group after cessation of DSS treatment. EcN-WT group displayed an improved state of weight loss in the first round of DSS administration compared with the DSS group but not in the following two rounds. The mice were sacrificed at Day 43 to investigate the shortening of colon lengths in order to examine the efficacy of EcN-Elafin in protecting against colitis. EcN-Elafin group displayed significantly alleviated shortening of colons compared with the DSS group and EcN-WT group, suggesting that EcN-Elafin could effectively protect against DSS-induced colitis in mice. In comparison, the DSS group and the EcN-WT group both displayed significantly shortened colon length compared with the PBS control group (Figure 1D).

We also assessed the severity of colonic inflammation in each group using histological analysis. Common histological changes include ulcer formation, colonic crypt loss, and immune cell infiltration. As shown in Figure 1E, all three groups that received DSS treatment displayed significantly elevated histological score than the healthy control group, suggesting more severe inflammatory responses in the colon. EcN-Elafin group showed significantly decreased histopathological score compared with DSS group. As shown in the HE staining of the colonic sections, mice in EcN-Elafin group showed decreased infiltration of immune cells and better colonic crypt structure preservation compared with the DSS group (Figure 1F).

### EcN-Elafin Enhances the Epithelial Barrier and Resolves Inflammation in the Gut

**Figure 2:**
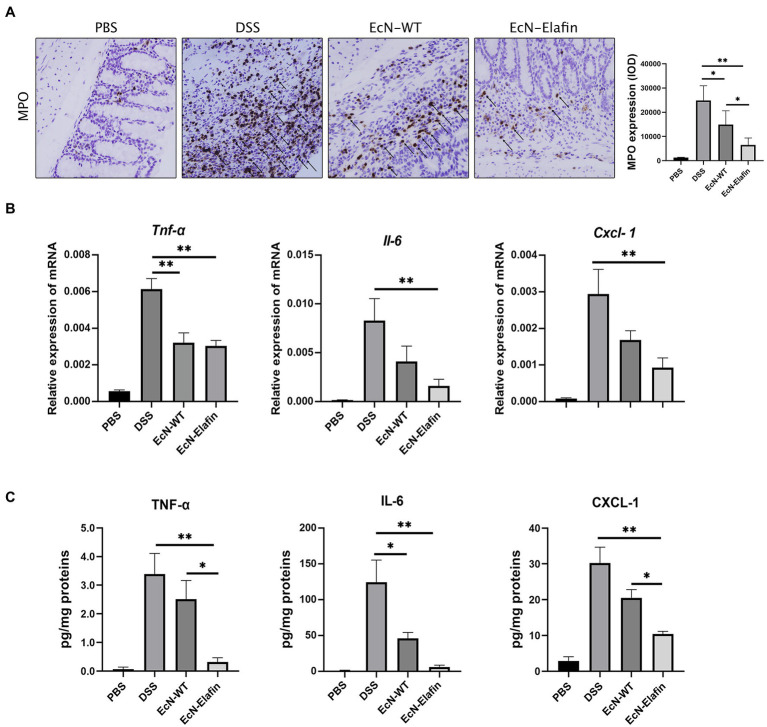
EcN-Elafin downregulated the expression of proinflammatory mediators in the colonic tissues from DSS-treated mice. **(A)** The expression of MPO in the colons from each group. Arrows indicate MPO positive cells in each group. **(B)** Relative mRNA expression and **(C)** protein levels of TNF-α, IL-6, and CXCL-1 in conic tissues from each group. 2^ΔCT^ method was used to calculate relative expression of mRNA. Protein levels were presented as pg/mg of proteins. *n* = 6–8 in each group. The data are presented as the mean ± SEM. ^*^p < 0.05; ^**^p < 0.01.

To investigate the possible mechanism underlying the protective effect of EcN-Elafin, we analyzed the gene expression and protein levels of proinflammatory cytokines and chemokine in the colonic segments in four groups. As shown in Figures 2B,C, the protein levels of TNF-α, CXCL-1, and IL-6 were significantly downregulated (*p* < 0.05) in the EcN-Elafin group compared with DSS group. It is worth noting that the expression of CXCL-1 (functional IL-8 homologue in mice) was significantly decreased in the EcN-Elafin group compared with the wild-type EcN group. Staining for markers of neutrophils (MPO) was quantified in mouse colon tissue (Figure 2A). MPO expression in the colon was increased after 3 rounds of DSS challenge and was significantly reduced by treatment with EcN-Elafin.

**Figure 3:**
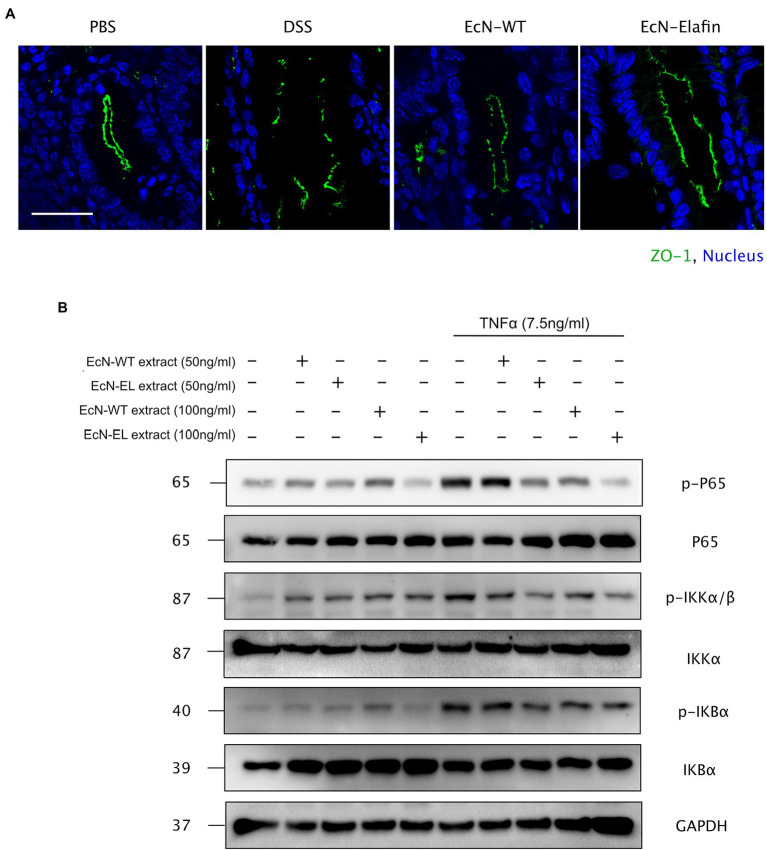
EcN-Elafin enhanced the intestinal epithelial barrier in mice with colitis. **(A)** Representative photo of immunofluorescence staining for the epithelial tight junction protein ZO-1 in mouse colonic tissues. Scale bar = 20 μm. **(B)** Caco-2 cells were cultured and then stimulated with recombinant TNF-α (7.5 ng/ml) in the presence or absence of either EcN-WT or EcN-Elafin bacterial extracts for 30 min. Then, the cell lysates were analyzed for p-IκBα, IκBα, p-IKKβ, IKKβ, p-P65, P65, and GAPDH expression by Western blotting.

Considering the significant role of tight junction proteins in maintaining intact intestinal epithelial barrier function, we therefore assessed whether tight junction integrity in colon was affected by administration of EcN-Elafin. The expression of occludin, claudin-1, and claudin-2 were unaffected by administration of EcN-WT and EcN-Elafin (data not shown), while expression and distribution of ZO-1 were markedly altered. As shown in Figure 3A, the expression of ZO-1 was significantly reduced in colons of the DSS group compared with PBS group and the distribution pattern of ZO-1 was patchy and intermittent, suggesting that tight junction integrity was impaired in mice challenged with DSS. In contrast, the localization of ZO-1 in mice treated with EcN-Elafin almost returned to normal.

Because the expression of the proinflammatory cytokine TNF-α along with the expression and distribution of the ZO-1 protein is partially under the regulation of NF-κB, the effect of EcN-Elafin on the activity of this transcription factor was then investigated *in vitro*. Previous studies have shown the damaging effect of TNF-α on intestinal barrier function is mediated through activation of NF-κB signaling. Thus, different concentrations of bacterial extracts (50 ng/ml and 100 ng/ml) were added simultaneously with or without 7.5 ng/ml TNF-α to Caco-2 cells for 30 min. The activation level of NF-κB signaling in Caco-2 cells was further analyzed by Western blotting. As shown in Figure 3B, phosphorylation of P65 was inhibited by both EcN-WT and EcN-Elafin extracts at concentration of 100 ng/ml. When bacterial extracts were added to the medium at concentration of 50 ng/ml, only EcN-Elafin was able to inhibit the phosphorylation of NF-κB P65 effectively. Furthermore, both EcN-WT and EcN-Elafin inhibited TNF-α induced phosphorylation of IKKα/β in Caco-2 cells in a dose-dependent manner. EcN-Elafin also slightly suppressed the phosphorylation of IKBα. These results demonstrate that EcN-WT and EcN-Elafin could inhibit NF-κB signaling in Caco-2 cells, and EcN-Elafin displays a stronger downregulating effect on NF-κB signaling than EcN-WT.

### EcN-Elafin Modulates the Gut Microbiota in Mice With DSS-Induced Colitis

**Figure 4:**
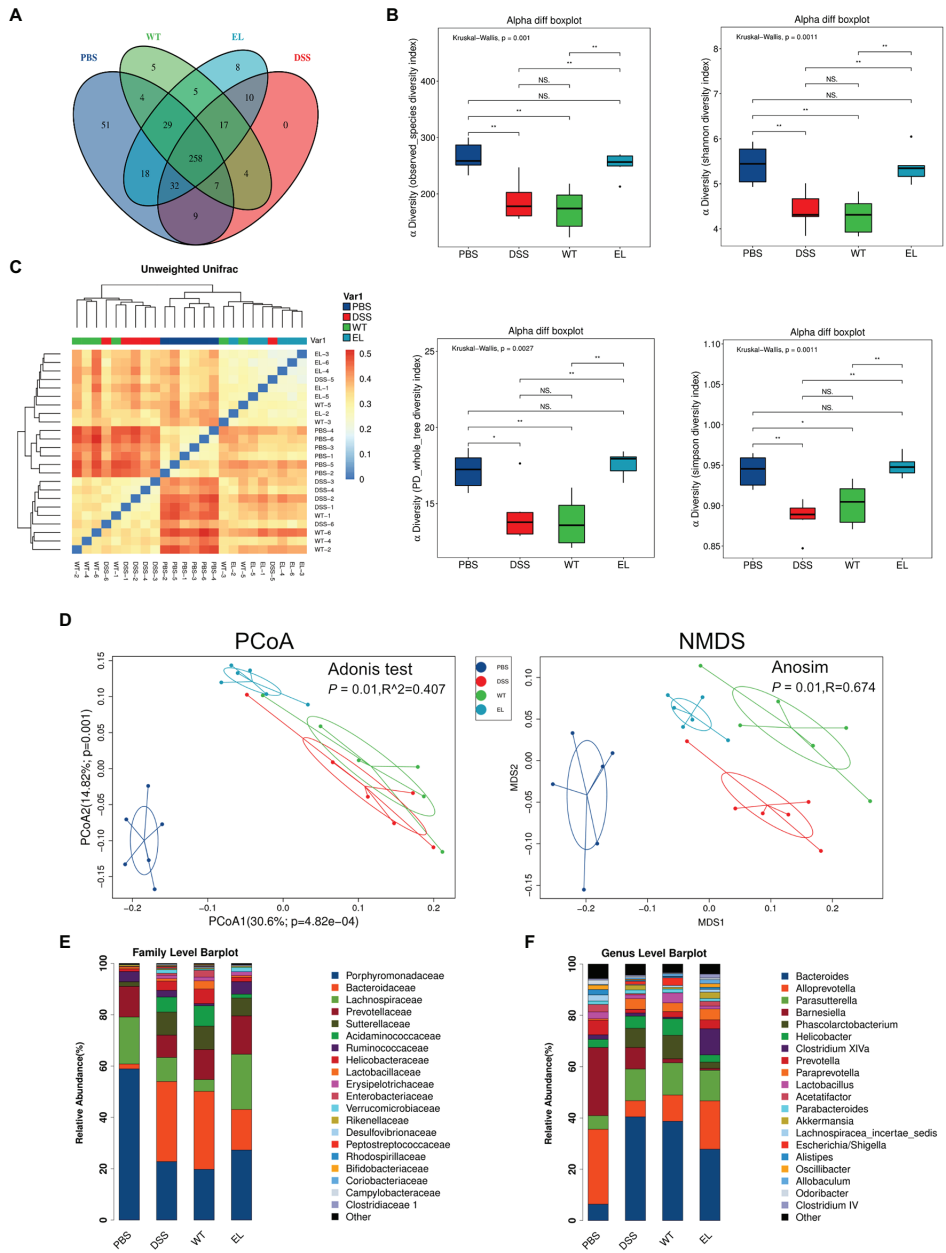
EcN-Elafin restored the disturbed gut microbiota in DSS-induced chronic colitis mice. **(A)** The Venn diagram exhibiting the overlaps of OTUs from four groups; **(B)** α-diversity was calculated by the observed-species diversity, PD-whole-tree diversity, and Shannon and Simpson indices. ns: not significant. ^*^p < 0.05 and ^**^p < 0.01 by Mann–Whitney test; **(C)** Heatmap with clusters displaying the results of unweighted Unifrac analysis among four groups **(D)** β-diversity analysis of the gut microbiota of each group. Ordination plots based on the PCoA and NMDS analysis using unweighted UniFrac. **(E**,**F)** Relative abundances of bacterial taxa at the family and genus level. *n* = 6 per group.

**Figure 5:**
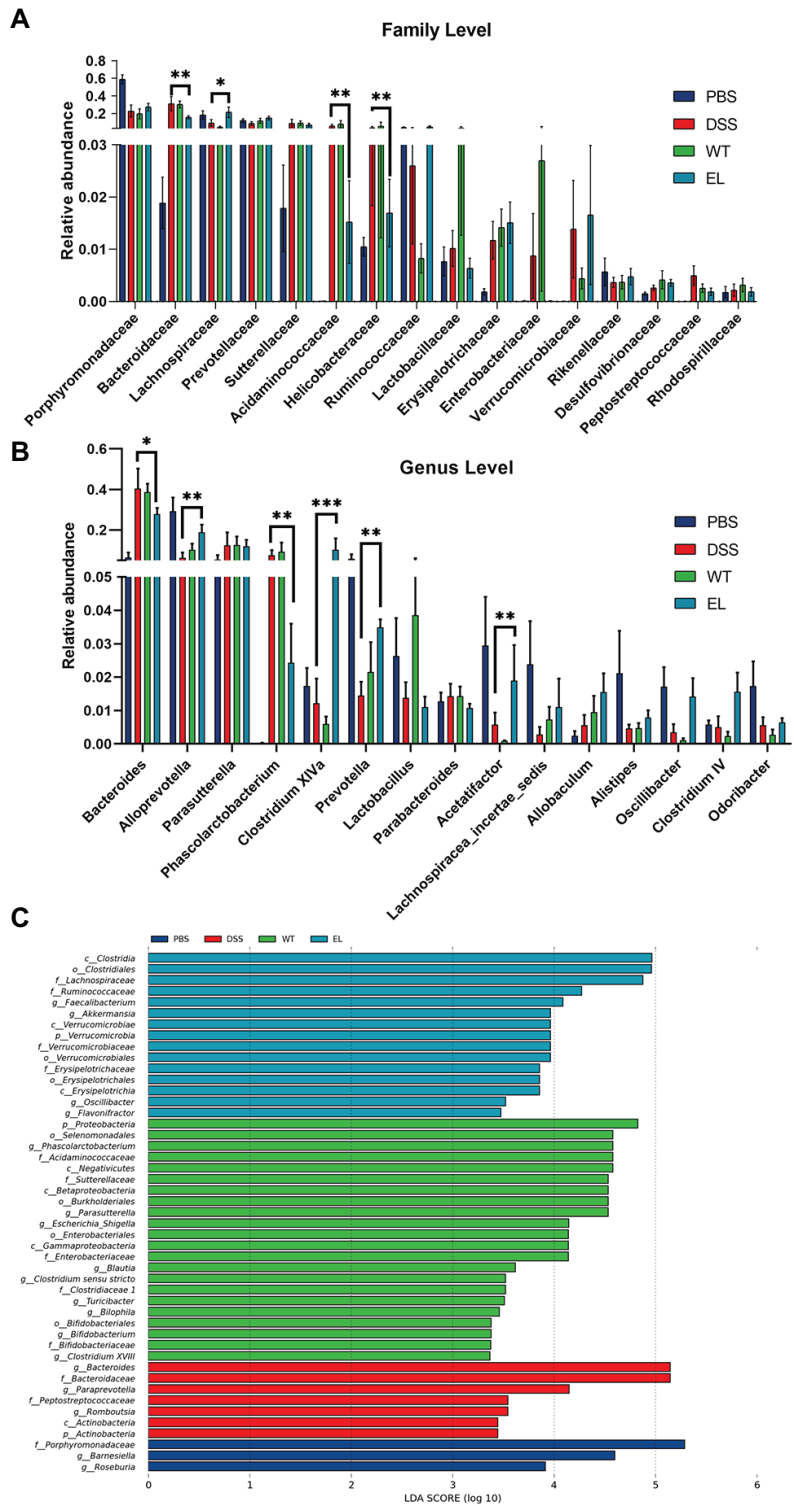
Effect of EcN-Elafin on the bacterial composition in the gut of DSS-induced chronic colitis mice. **(A)** Relative abundances of a common set of microbial families; **(B)** Relative abundances of a common set of microbial genera; **(C)** Differences in microbiota in each group were calculated by LDA effect size (LEfSe). *n* = 6 per group. Kruskal-Wallis test was used with a statistical significance cutoff of *p* < 0.05 and the threshold of LDA score is 2. p, phylum; c, class; o, order; f, family; and g: genus.

The above results and previous studies suggesting the antimicrobial properties of Elafin led to the hypothesis that EcN-Elafin may alter the gut microbiota composition. Therefore, we characterized the microbial composition of colonic content collected from mice in each group utilizing 16S ribosomal DNA (rDNA) gene sequencing analysis. The numbers of OTUs identified in each group were as follows: 408 in PBS group, 329 in EcN-WT group, 377 in EcN-Elafin group, and 337 in DSS group (overlaps of OTUs were shown in Figure 4A). As shown in Figure 4B, analysis of α-diversity using the observed-species diversity, PD-whole-tree diversity, phylogenetic diversity, and Shannon and Simpson indices showed that DSS treatment reduced species richness significantly (*p* < 0.05) of bacteria in the gut, and no changes in α-diversity were found between the EcN-WT group and the DSS group. Notably, the EcN-Elafin group showed significantly restored bacterial species richness compared with the EcN-WT group (*p* < 0.05). β-diversity was analyzed by unweighted UniFrac metrics. As shown in the unweighted unifrac heatmap analysis, the similarity of composition of gut bacteria community is closer between PBS group and EcN-Elafin group (Figure 4C). The microbial composition of EcN-Elafin group is significantly different from EcN-WT group and DSS group, as presented by the distinct clustering pattern in principal coordinates analysis (PCoA) plot and non-metric multidimensional scaling (NMDS) plot. Collectively, the results suggest that EcN-Elafin administration can effectively restore the disturbed gut microbiota composition induced by DSS administration. As shown in Figure 4E, the most abundant families in the fecal sample collected from each group were *Porphyromonadaceae*, *Bacteroidaceae*, and *Lachnospiraceae*. At the genus level, the fecal microbiota of the PBS group was dominated by *Alloprevotella*, *Barnesiella*, and *Bacteroides*, while the other three groups were dominated by *Bacteroides*, *Parasutterella*, and *Alloprevotella* (Figure 4F). At the family level (Figure 5A), compared with the DSS group, the EcN-Elafin group showed an increase in *Lanchnospiracea* and a decrease in *Bacteroidaceae*, *Acidaminococcaceae*. At the genus level (Figure 5A), the EcN-Elafin group showed an increase in *Alloprevotella*, *Clostridium* cluster XIVa, *Prevotella*, and *Acetatifactor* and a decrease in *Bacteroides* and *Phascolarctobacterium* compared with DSS group. We performed linear discrimination analysis (LDA) using the LDA effect size (LEfSe) algorithm to identify operational microbial taxa that were differentially abundant in each group. After EcN-Elafin treatment, relative abundances of *Clostridiales*, *Lachnospiraceae*, and *Ruminococcaceae* were significantly increased (Figure 5C).

### EcN-Elafin Alters SCFA Abundance in Mice With DSS-Induced Colitis

**Figure 6:**
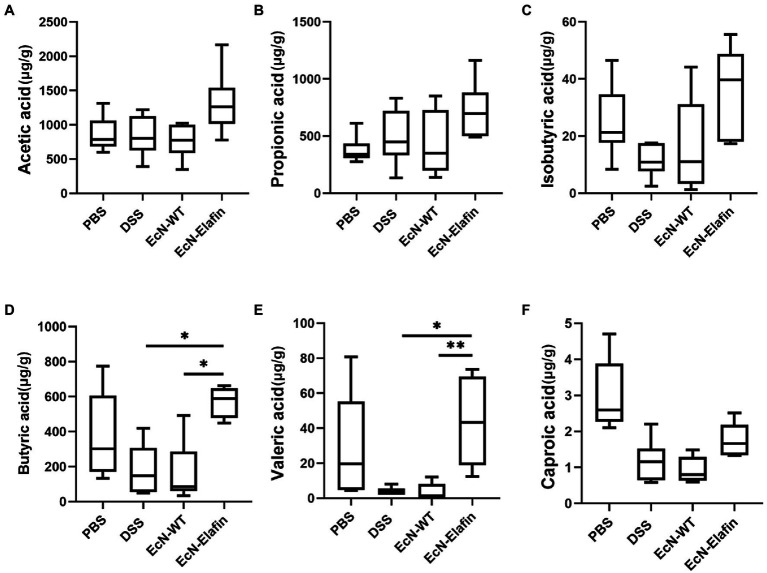
EcN-Elafin increased the levels of SCFAs in the gut of DSS challenged mice. Levels of SCFAs in fecal samples, including **(A)** acetic acid, **(B)** propionic acid, **(C)** isobutyric acid, **(D)** butyric acid, **(E)** valeric acid, and **(F)** caproic acid in each mouse were determined. *n* = 6 in each group. ^*^p < 0.05; ^**^p < 0.01.

Short-chain fatty acids (SCFAs) are bacterial metabolites from dietary components. Considering the protective effect of EcN-Elafin on intestinal flora composition and the effect of SCFAs on the immune response, we then measured the SCFAs in the colonic content collected from all four groups (Figures 6A-F). Concentrations of SCFAs in DSS group showed a tendency to decrease compared with PBS group but the difference is not statistically significant. When compared with the DSS group, EcN-Elafin significantly elevated the levels of butyrate and valerate in the gut lumen (Figures 6D,F). Other analyzed SCFAs concentration showed no significant difference among four groups.

## Discussion

In this study, an engineered probiotic that is capable of expressing recombinant Elafin is successfully constructed. Administration of this modified probiotic during the induction of chronic colitis in mice was able to ameliorate inflammation in the gut, and this therapeutic effect can be attributed to the probiotic nature of EcN and the anti-inflammatory and inhibition of proteases properties of Elafin. Engineered EcN colonized in the colon can enhance the colonic epithelial barrier, decrease the level of proinflammatory cytokines and enhance abundances of other probiotics, and alter the SCFAs concentration in the gut of DSS challenged mice.

Elafin is a small molecular weight protein that specifically inhibits NE and PR-3 (Verrier et al., 2012). Previous studies have found that Elafin is a multifunctional host defense protein in the respiratory tract which protects the host against a variety of pathogenic microbes (bacteria, fungi, and viruses) (Small et al., 2017). In addition, a recently completed clinical trial has proved the safety of Elafin administrated subcutaneously in both healthy volunteers and patients with pulmonary arterial hypertension under the dosage ranging from 0.03 to 0.18 mg/kg (NCT03522935). Compared with other anti-inflammatory molecules used to treat colitis, Elafin is a human endogenous protein and is expressed in human intestinal mucosa; thus, fewer side effects may be expected when applied to treating IBD. Our results are in line with the previous studies that Elafin can exert potent anti-inflammatory properties at the colonic mucosa (Motta et al., 2011, 2012). However, whether Elafin is able to modify the gut microbiota is poorly understood.

Tight junctions regulate intercellular permeability, and their localizations have been identified as critical factor in maintaining the integrity of the epithelial barrier. Alterations in tight junction integrity are associated with increased susceptibility to IBD (Su et al., 2013). Previous study *in vitro* showed myosin light chain kinase (MLCK) inhibition increased barrier function and stabilized ZO-1 but did not affect the expression of occludin and claudin-1 (Yu et al., 2010). We show here EcN-Elafin administration restricts paracellular permeability by regulating the expression and distribution of tight junction protein ZO-1 (Figure 3A). However, measurements of transepithelial electrical resistance (TEER) in Caco-2 monolayer model should be considered in a future study with focus on the effect of purified recombinant Elafin on colonic epithelial barrier function and the MLCK signaling-mediated regulation of ZO-1 expression.

UC is characterized by unremitting inflammation in the colon and increased plasma levels of proinflammatory cytokines and chemokines including TNF-α and IL-8 (Rana et al., 2014; Zhang et al., 2017). TNF-α has been widely used as a damage factor in Caco-2 cells to mimic the intestinal barrier injury in UC patients (He et al., 2012; Subramanian et al., 2018). In the present study, Caco-2 cells challenged with TNF-α were incubated with either EcN-WT or EcN-Elafin extracts to investigate the effect of EcN-Elafin extracts on the activation level of NF-κB signaling. Our results demonstrate that EcN-WT extracts are able to downregulate the activity of NF-κB signaling and confirms the previously reported probiotic effects of EcN through inhibiting NF-κB pathway (Guo et al., 2019). However, EcN-Elafin was more efficient than EcN-WT at downregulating NF-κB signaling in a dose-dependent manner.

The human gastrointestinal tract is home to trillions of microorganisms. Accumulating studies are revealing that gut microbiota and their metabolites significantly impact the health of the host and may have played an essential role in the etiology of IBD (Lavelle and Sokol, 2020; Van Treuren and Dodd, 2020). It is clear that reductions in gut microbiome diversity are correlated with IBD severity (Lee and Chang, 2021). Our data demonstrated that the gut microbial composition in EcN-Elafin group was significantly different from that in the DSS group but was similar to that of the PBS group, as evidenced by both α and β-diversity analyses of gut bacterial community composition. In addition, mice in EcN-Elafin group showed restored disturbed gut microbiota when challenged by DSS, with a reduction in opportunistic pathogens belonging to *Bacteroidaceae*, *Helicobacteraceae* families and enhanced growth of beneficial microbes belonging to *Lachnospiraceae*, *Clostridium* cluster XIVa, and *Alloprevotella*. In addition, increased relative abundance of *Prevotella* and *Phascolarctobacterium* were also observed in EcN-Elafin group when compared with DSS group. *Prevotella* is a large genus with high species diversity between strains; thus, it is difficult to predict how *Prevotella* will function in different intestinal ecological settings (Ley, 2016; Larsen, 2017; Gálvez et al., 2020). *Phascolarctobacterium* belonging to *Acidaminococcaceae* can use the succinate pathway to produce SCFAs, including acetate and propionate (Wu et al., 2017). A recent study has shown that *Phascolarctobacterium* could reduce the luminal concentration of succinate, which is a crucial metabolite for the growth of C. *difficile*, and therefore prevent the C. *difficile* infection (Nagao-Kitamoto et al., 2020).

However, no significant difference between the EcN-WT group and the DSS group in terms of α and β-diversity of the gut microbiome. One explanation for this result could be that we used a different experimental mouse model and bacterial administration scheme. Therefore, the experimental colitis model used may influence the gut microbiota composition in mice and its reaction to treatment. Hence, the further applicability of EcN-Elafin for treating IBD patients requires future studies in other colitis models (e.g., IL-10 knockout and TNBS-induced colitis). However, this study provides convincing evidence that EcN-Elafin is able to restore gut microbial ecology, and this effect is mainly attributed to Elafin. To the best of our knowledge, our results provide evidence, for the first time, that Elafin could exert a protective effect against colitis through modifying the intestinal microbiota composition.

Short-chain fatty acids (SCFAs) are derived from bacterial fermentation of dietary fibers in the colonic lumen. The SCFAs butyrate, propionate, and acetate could promote intestinal epithelial barrier function and regulate the mucosal immune system in the host (Vinolo et al., 2011; Yang et al., 2019). Exposure of peripheral blood mononuclear cells (PBMCs) such as macrophages and neutrophils to SCFAs inhibits inflammatory cytokine production (Usami et al., 2008; Vinolo et al., 2011). A mouse model of intestinal inflammation suggests that butyrate plays a pivotal role in regulating immune activity *in vivo* (Furusawa et al., 2013; Tye et al., 2018; Hao et al., 2021). In this study, we have quantified SCFAs in the fecal samples collected in each group. Concentrations of SCFAs in DSS group showed a tendency to decrease compared with PBS group although the difference is not statistically significant. The mechanism of DSS-induced colitis mouse model still remains to be elucidated. DSS can specifically cause damages to colonic epithelia and sequentially cause dysbiosis in the gut lumen. But the changes of SCFAs in fecal samples after DSS administration remain to be elucidated. In the present study, significantly increased concentrations of butyrate and valerate in fecal samples collected from mice in the EcN-Elafin group has been observed. In addition, *Lachnospiraceae* and *Clostridium* cluster XIVa which are capable of producing butyrate were highly abundant in EcN-Elafin group. A previous study has shown that the butyrate-producing bacteria *Clostridium* cluster XIVa could influence the number and function of regulatory T cells (Tregs) in the colon and therefore protect against colitis (Atarashi et al., 2011). Thus, further experiments utilizing fecal microbiota transplantation (FMT) methods could be performed to investigate the effects of orally administrated EcN-Elafin on Treg immunity in the colonic lamina propria.

All the findings together suggest that EcN-Elafin is able to (i) enhance the intestinal epithelial barrier, (ii) promote the resolution of inflammation, (iii) reshape the gut microbial community composition, and (iv) elevate the concentration of SCFAs in gut lumen to alleviate colitis. However, a limitation of this study is that the experiment was conducted in a rodent model. The intestinal microbiota compositions of humans are not identical to those of mice. Therefore, the observed effects of EcN-Elafin on the composition of intestinal microbiota must be confirmed by further clinical trials.

In conclusion, our data suggest that delivering Elafin to the colonic epithelial surface with the help of the probiotic bacteria EcN can effectively ameliorate the inflammation in a chronic DSS-induced mouse model. This study suggests that there may be a potential clinical application for EcN-Elafin in treating IBD. This approach may offer a cost-effective treatment method for IBD.

## Data Availability Statement

The original contributions presented in the study are included in the article, further inquiries can be directed to the corresponding authors.

## Ethics Statement

The animal study was reviewed and approved by Experimental animal ethics committee of the First Hospital of Peking University.

## Author Contributions

GT and ZL contributed equally to this work and should be considered joint first authors. YD, WW, and HW should be considered joint corresponding authors. All authors contributed to the article and approved the submitted version.

## Funding

This study was supported by the Beijing Natural Science Foundation (no. 7202209) and the National Natural Science Foundation of China (no. 81800492) awarded to GT. The 13th Five-Year Plan for National Key R&D Program of China (no. 2018YFC1705404) awarded to HW.

## Conflict of Interest

The authors declare that the research was conducted in the absence of any commercial or financial relationships that could be construed as a potential conflict of interest.

## Publisher’s Note

All claims expressed in this article are solely those of the authors and do not necessarily represent those of their affiliated organizations, or those of the publisher, the editors and the reviewers. Any product that may be evaluated in this article, or claim that may be made by its manufacturer, is not guaranteed or endorsed by the publisher.

## Footnotes

^1^ http://rdp.cme.msu.edu
